# Multi-temporal ecological niche modeling for bird conservation in the face of climate change scenarios in Caatinga, Brazil

**DOI:** 10.7717/peerj.14882

**Published:** 2023-02-27

**Authors:** Gabriela Silva Ribeiro Gonçalves, Pablo Vieira Cerqueira, Daniel Paiva Silva, Letícia Braga Gomes, Camila Ferreira Leão, André Felipe Alves de Andrade, Marcos Pérsio Dantas Santos

**Affiliations:** 1 Laboratório de Biogeografia da Conservação e Macroecologia, Universidade Federal do Pará, Belém, Brazil; 2 Departamento de Biologia, Instituto Federal Goiano, Urutaí, Goiás, Brazil; 3 Instituto de Ciências Biológicas, Universidade Federal de Goiás, Goiânia, Goiás, Brazil

**Keywords:** Climatic stability, Conservation policy, Dry forest, Extinctions, Gap analysis, Protected areas

## Abstract

**Background:**

Global shifts in climatic patterns have been recorded over the last decades. Such modifications mainly correspond to increased temperatures and rainfall regime changes, which are becoming more variable and extreme.

**Methods:**

We aimed to evaluate the impact of future changes in climatic patterns on the distribution of 19 endemic or threatened bird taxa of the Caatinga. We assessed whether current protected areas (PAs) are adequate and whether they will maintain their effectiveness in the future. Also, we identified climatically stable areas that might work as refugia for an array of species.

**Results:**

We observed that 84% and 87% of the bird species of Caatinga analyzed in this study will face high area losses in their predicted range distribution areas in future scenarios (RCP4.5 and RCP8.5, respectively). We also observed that the current PAs in Caatinga are ineffective in protecting these species in both present and future scenarios, even when considering all protection area categories. However, several suitable areas can still be allocated for conservation, where there are vegetation remnants and a high amount of species. Therefore, our study paves a path for conservation actions to mitigate current and future extinctions due to climate change by choosing more suitable protection areas.

## Introduction

Global shifts in climatic patterns have been recorded over the last decades. They consist mainly of increased temperatures and changes in rainfall regimes, which have become more variable and extreme (Bindoff et al., 2013; Marzeion, Jarosch & Gregory, 2014; Meier et al., 2014). Severe climate changes, which could result in increased aridity, are predicted for several tropical biomes (Salazar, Nobre & Oyama, 2007; Marengo, Torres & Alves, 2017; Anjos & Toledo, 2018). In South America, for instance, savannas might replace some forested locations, and semi-desert areas might expand throughout the Northeast region of Brazil (Oyama & Nobre, 2003). Such changes imply significant climate alterations soon, causing great shifts in interactions among biological communities, such as the dynamics of predation, herbivory, competition and hostplant associations (Newbold et al., 2015; Pecl et al., 2017). Once this process is unraveling quite rapidly, species might not have enough time to adapt to the novel environmental conditions, resulting in significant biodiversity loss and the disruption of ecological services (Nobre, Reid & Veiga, 2012; Pecl et al., 2017).

The maintenance of biodiversity and ecosystem service depends mainly on the ability of organisms to adapt to these future climate changes (Hughes, 2000). Therefore, species exposed to fast climatic alterations will face a higher extinction risk (Parmesan & Yohe, 2003; Foden et al., 2008; Nogués-Bravo et al., 2010). However, responses to the effects of these changes are not uniform and vary from one species to another and between different taxonomic groups (Jetz, Wilcove & Dobson, 2007; Bellard et al., 2012; Şekercioĝlu et al., 2012). Therefore, species are expected to move throughout the geographical space towards isotherms that ensure their optimal development, maintaining the best possible functioning of their physiological processes (Forrest, 2015; Rafferty, Caradonna & Bronstein, 2015). In this way, species can respond to climate change by adapting to new conditions, changing their geographic distribution, or becoming extinct (Jezkova & Wiens, 2016; Lenoir & Svenning, 2015).

This change in species distribution might affect the probability of these species persisting in areas allocated for conservation, thus, representing a massive challenge for developing approaches that protect several species (Hannah et al., 2013; Lemes & Loyola, 2013). Currently, protected areas (PAs) are the pillars for the protection and maintenance of biodiversity. However, climate change is rarely considered in the establishment of PAs (Jones et al. , 2017), which can result in inadequate protection of biological diversity (Araújo et al., 2011; Baldi et al. , 2017; Carvalho et al., 2022). Therefore, evaluating the effectiveness of protected areas is essential since suitable and enough areas for conservation might become insufficient or unsuitable shortly (Lemes, Melo & Loyola, 2014). Thus, climate change creates a significant challenge for developing systematic planning for species conservation.

The Caatinga (dry forests in Northeastern Brazil) is among the most vulnerable Brazilian domains to climate variability extremes. Global and regional predicted climate change scenarios show that the region will be affected by reduced rainfall and increased temperatures, contributing to increased aridity and subsequent desertification (Marengo, Torres & Alves, 2017; MMA, 2007). Approximately 94% of the Caatinga region is under moderate to high susceptibility to desertification (Vieira et al., 2015; Vieira et al., 2020). In addition, temperatures in the area are projected to increase by 4 °C in the RCP 8.5 scenario (Marengo, Torres & Alves, 2017). Thus, the combination of reduced rainfall, increased temperature, soil degradation, and desertification can make this region one of the world’s most vulnerable to climate change (IPCC, 2014).

Furthermore, the Caatinga has undergone a rapid and sudden environmental alteration derived directly or indirectly from human activities (Andrade et al., 2005; Marinho et al., 2016; MMA, 2007). Original landscapes in this domain have become highly heterogeneous mosaics due to high habitat loss and fragmentation. Approximately 46% of the Caatinga is estimated to have already been deforested (MMA, 2011). Such environmental degradation and the consequent habitat loss might reduce even more options for conservation, thus, restricting essential conservation areas in a future climate scenario. This domain has the lowest number of protected areas and the most limited protected extension among all Brazilian domains, corresponding to only 9% of its territory (Brasil, 2020). Due to all these factors, the Caatinga represents an ideal tropical model for studies on the effects of climate change on species distribution. The region is one of the world’s largest and most biodiverse tropical drylands (Silva, Leal & Tabarelli, 2017; Araújo et al., 2022), owning 554 bird species (Araújo et al., 2022). Regarding organisms sensitive to environmental changes, birds serve as bioindicators. They are considered critical organisms for maintaining ecological balance due to their ability to disperse seeds (Viana et al., 2016; Pauw , 2019; González-Varo et al., 2021), insect population control and pollination (Whelan, Wenny & Marquis, 2008). Currently, Caatinga birds already suffer from the severe effects during years of intense drought, with increased vulnerability, population declines and even local extinctions, mainly of pollinators (Toledo-Lima & Pichorim, 2020). Thus, this group is an excellent model for understanding the effects of future climate change.

Considering this scenario, our objective was to understand the effects of climate change predicted for the future on the distribution of endemic bird species in the Caatinga, using for this the methodology of ecological niche modeling. Additionally, we aimed to determine which areas are the richest and most stable under different climate scenarios, assessing whether the current PAs are adequate and whether they maintain their effectiveness in the future.

## Methods

### Study area

Caatinga covers 844,453 km^2^ and is the only domain restricted to Brazil. This Brazilian biome is part of a complex of forest vegetation types with unique characteristics throughout the Neotropics, named Seasonally Dry Tropical Forests (SDTFs; Pennington, Lewis & Ratter, 2006). This biome extends from 2°54′S to 17°21′S latitudes and occupies the Brazilian northeast and parts of Minas Gerais (Andrade-Lima, 1981; Paim & Franca-Rocha, 2009; Tabarelli & Da Silva, 2003). It is considered one of the most complex ecoregions worldwide (Nimer, 1972; Reis, 1976). The Caatinga is situated at the convergence zone of several highly unstable air masses. Also, this biome is marked by Brazil’s strongest insolation, high thermal averages (26–29 °C), low relative humidity percentages, and scarce and irregular rainfalls (annual 250–800 mm). Finally, it is a reasonably seasonal domain, with rains distributed through a short period of the year (2–3 months) and extensive periods of cyclic droughts (Nimer, 1972; Reis, 1976).

**Figure 1:**
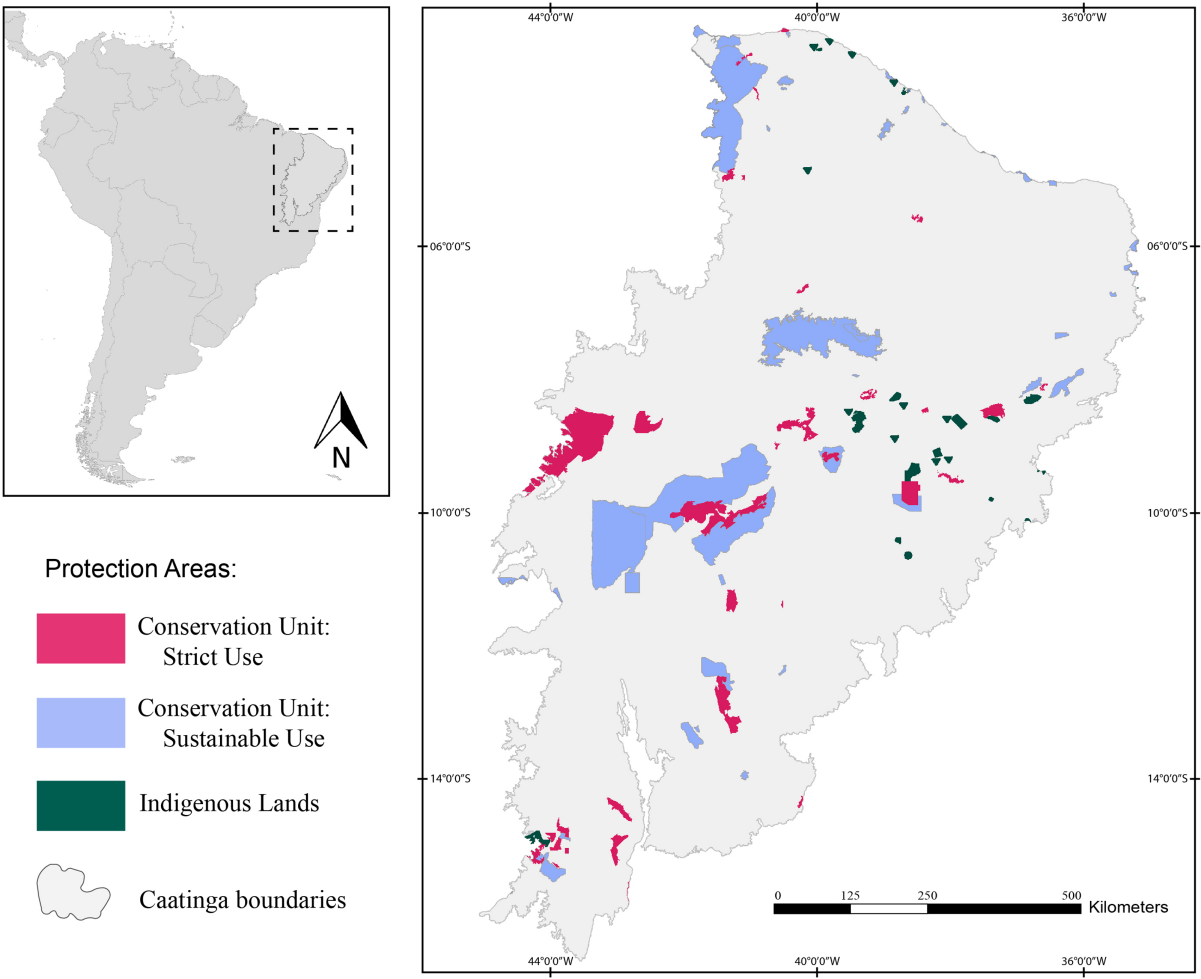
Map of the study area showing the location of the Caatinga domain. The areas in pink are Strictly Protected Areas, blue areas represent Sustainable Use Areas, and areas in green correspond to Indigenous Lands. Broadview of the location of the Caatinga domain in South America. Location of Protection Areas in the Caatinga domain. Shapefile provided by MMA (Ministério do Meio Ambiente do Brasil - Ministry of the Environment).

Herein, we assessed 127 protection areas (PAs) distributed throughout the Caatinga domain (Fig. 1). Of those, 38 are Strictly Protected Areas (SPA), 52 are Sustainable Use Areas (SUA), and 37 are Indigenous Lands (IL).

### Target species and occurrence dataset

In this study, we selected 19 bird taxa from Caatinga that can be more sensitive to climate change, all the bird taxa included in the analysis were based in the following criteria: restricted distribution (endemic, near-endemic) and/or threatened status by The International Union for Conservation of Nature red list - IUCN (see Table S1 and Table S2 in Supporting Information).We gathered distribution data for each taxon based on literature reports, online databases Species Link (http://splink.cria.org.br/), Xeno-canto (http://www.xeno-canto.org/), Wikiaves (http://www.wikiaves.com.br/), Vertnet (http://www.vertnet.org/index.html), Ebird (http://www.ebird.org/), specimens held in Museu Paraense Emílio Goeldi, and personal data (PVC, GSRG, MPDS). All the occurrence records were adequately checked, undergoing a strict analysis process by bird experts. Therefore, we obtained a total of 1,523 unique occurrences (Table S1). We followed the taxonomy adopted by the Brazilian Committee of Ornithological Records (Pacheco et al., 2021). We controlled sampling bias on georeferenced data by removing duplicate records and leaving a single record per pixel. We used a thinning technique using the package spThin (Aiello-Lammens et al. , 2015) to reduce autocorrelation in occurrence data. Afterward, we used Moran’s I and variograms that minimize spatial autocorrelation to define the thinning distance (Andrade, Velazco & De Marco, 2020).

### Environmental data

For the current scenario, we used 19 environmental variables available at WorldClim version 2.0 (http://www.worldclim.org/current; Hijmans et al., 2005) at a scale of ∼5 km^2^. To avoid collinearity between the climate variables, we performed a principal component analysis (PCA) to reduce the dimensions, thus using as predictors of the response functions the axes that explained 95% of the total variance (De Marco & Nóbrega, 2018). To maintain the dimensionality of climate data over time, the coefficients obtained from the PCA performed with present climate data were used to compute scores for future climate data (Sillero & Barbosa, 2021). For future scenarios, we used the same climatic variables from the general circulation models (Atmosphere-Ocean General Circulation-AOGCMs) available for 2070. We performed a cluster analysis to select the AOGCMs proposed by Varela, Lima-Ribeiro & Terribile (2015) because it aims to maximize uncertainties between climate models. The selected AOGCMs were: CCSM4 (CC), HadGEM2-AO (HD), IPSL-CM5A-LR (IP), MIROC-ESM (#) (MR), MRI-CGCM3 (MG). We used two representative concentration pathways (RCP4.5 and 8.5) to assess the effect of climate change on target species distribution.

### Data partition and modeling proceedings

We used the “leave-one-out” (LOO) method proposed by Pearson et al. (2007) to predict the distribution of species with less than 20 occurrence records so that we could partition the occurrences and evaluate whether the produced distributions were reliable. The LOO method is recommended when modeling species with a small number of occurrences since it indicates if they are not within the modeled distribution area (Lima-Ribeiro & Diniz-Filho, 2012). For our species the lowest number of occurrence records was six, in the literature it is estimated that the minimum number of occurrence records should be three to five in order to have reliable predictions (Almeida, Côrtes & De Marco, 2010; Lima-Ribeiro & Diniz-Filho, 2012). Thus, we jackknifed the observations to produce occurrences subsets with n-1 occurrences for our data input. After, we used these subsets to predict the distribution of the species. The occurrence record that was left out was used to evaluate the goodness-of-fit of the models. For distribution predictions to be considered reliable, *p* values must be less than 0.05, indicating that there would be no relevant sampling bias in the occurrence data set used to model species distributions. Conversely, *p*-values greater than 0.05 indicate an unreliable outcome.

For species with more than 20 occurrences, models were generally evaluated by cross-validation, considering the independent data set. However, it is necessary to have some caution when maximizing the independence of training and test subsets (Roberts et al., 2017). Therefore, we have partitioned the occurrence data similarly to the *checkerboard* partition method (Muscarella et al., 2014; Valavi et al., 2019; Velazco et al., 2019), using the ENMTML R package (Andrade, Velazco & De Marco, 2020) available on GitHub (https://github.com/andrefaa/ENMTML). Data were geographically partitioned based on grids of different sizes. We then chose the grid size that maximized the independence and environmental similarity between the subsets. In this approach, both subsets are used first to adjust the model and then later to evaluate the model.

Many algorithms are used to predict species distribution based on different statistical approaches and data inputs. Algorithm performance varies depending on the modeling condition (Norberg et al. , 2019; Qiao, Soberon & Peterson, 2015) and is one of the primary sources of model uncertainty (Thuiller et al., 2019). Thus, multiple algorithms allow us to identify the most suitable species and report model uncertainty (Norberg et al. , 2019; Thuiller et al., 2019). We used three algorithms (Maxent, SVM, and random forest) encompassing a range of statistical techniques for modeling species distributions. Maxent, SVM, and Random Forest were fitted using the R software v.3.5.1 (R Core Team, 2018), with the packages maxent v.0.1.2 (Phillips, 2017), kernlab v.0.9-25 (Karatzoglou et al., 2004) and randomForest v.4.6-12 (Liaw & Wiener, 2002), respectively. We evaluated models using the Jaccard Index (Jaccard, 1908). This index calculates similarity between the predictions and observations of the partitioned data, varies between 0 and 1 (Leroy et al., 2018). Index value closer to 1 means greater correspondence between predictions and observations, consequently, lower number of false positives and negatives, and better evaluated models (Leroy et al., 2018).

We made an ensemble forecast procedure to obtain the final ENM for each species (Araujo & New, 2007). The ensemble was obtained by calculating the arithmetic average of the suitability predicted by the best algorithms for each species. Therefore, the final model is the mean of the algorithms whose performances were greater than or equal to the algorithms’ average Jaccard value. For predicting the future distribution of birds, after performing the ensemble forecast to construct a single consensus model of the algorithms for a given GCMs, a new average of suitability values among the five GCMs was estimated to obtain a single future projection for each emission scenario. Subsequently, suitability maps (current and designed models) were transformed into binary maps based on the threshold values calculated from the Jaccard index (Pearson et al., 2007; Marco Jr & Siqueira, 2009). Finally, we performed pre- and post-processing modeling procedures with the ENMTML R package (Andrade, Velazco & De Marco, 2020; Mendes et al., 2020).

### Stacked species distribution models

We performed stacked-species distribution models (S-SDM) of all species to obtain species richness maps in the different scenarios in both current and future scenarios. This method has already been shown to be effective in several different situations (Distler et al., 2015; Guisan & Theurillat, 2000; Wisz et al., 2008). First, we stacked the current and future areas to obtain stability areas for each species and thus obtained a common (stable) area for each species in all predictions. Subsequently, we built a richness map with all stable areas by stacking stable regions of all species. For all stackings, we used the raster calculator tool of QuantumGis 2.8.

### Effectiveness of protected areas and conservation status of species

We used the shapefiles provided by the Ministry of Environment’s Cadastro Nacional de Unidades de Conservação do Ministério do Meio Ambiente (national registry of PAs; MMA, 2021) that contains Strictly Protection Areas (SPA), Sustainable Use Areas (SUA), and Indigenous Lands (IL) to represent the PAs. In total, the final dataset consisted of 127 PAs. We performed the analysis considering three types of PAs varying according to their protection levels: (1) Only SPA; (2) SPA + SUA; and (3) SPA + SUA + IL (Fagundes, Vogt & De Marco, 2016). The method employed in the gap analysis admits that species with restricted distribution must have all their occurrence area within PAs as they are more susceptible to extinction (Purvis et al., 2000).

However, widely distributed taxa should have at least 10% of their occurrence extension protected. Therefore, species with distribution areas covering less than 1,000 km^2^ must have 100% of their distribution protected, while those with more than 250,000 km^2^ must have at least 10% of their geographic distribution under protection. For species with intermediate distributional range sizes, a calculation is performed through interpolation using a logarithmic transformation, following the methods proposed by Rodrigues et al. (2003).

Finally, considering current and future scenarios, we classified species, regarding the targed of protection, as (1) protected (P) when the target percentage (≥90%) of species distribution size was within PAs; (2) partially protected (PP) when only one portion of the target percentage (<90% ≥ 20%) lays within PAs; (3) gaps (G) when only a percentage (<20%) of the target was within PAs, and (4) not protected (NP) when a 0 part of the target percentage was within the PAs (Frederico, Zuanon & De Marco, 2018; Velazco et al., 2022).

We evaluated the PA effectiveness using a null model approach: the ability of PAs to retain higher richness than would be expected by chance. For this, we compared the number of species within each PA with the estimated number of predicted species found within the PA according to a null model. This null model randomly allocated PAs within the Caatinga, maintaining their size, shape, and orientation (see Lemes & Loyola, 2013; Ferro et al., 2014; Ribeiro et al., 2016). In each run, we calculated the average value of species richness based on the cells encompassed by each PA for both current and future scenarios. PAs were effective if their observed species richness was greater than or equal to the null species richness obtained from randomizations in at least 95% of runs (*i.e.,* PAs with *p* < 0.05).

We also identified the representativeness degree of the species within the PAs in current and future scenarios (Araújo et al., 2011). We calculated the representativeness degree within the PAs network as the mean percentage overlap (MPO) for each species. MPO corresponds to the mean percentage of overlap between the PAs in the Caatinga biome and the given species occurring within the PAs. First, we calculated the spatial overlap of each cell with the PAs polygons. Then, we used null models to test whether the MPO of each species was statistically significant, considering the range size of the species. The MPO value observed for a given species was compared to the MPO values obtained from 1,000 random species with an equivalent size interval. This meant the same number of grid cells modeled for the species but extracted randomly within all study extent. Such a procedure allows us to identify whether the representativeness of a given species within the PAs considered, along with their MPO value, was significantly higher or lower than expected at random, considering a significance level of *p* < 0.05 (Araújo et al., 2011).

All five criteria of the IUCN Red List (criterian A–E) must be taken into account to assess the conservation status of species. However, in some cases, classification can also be done if at least one of the criteria is considered (UNEP-WCMC, 2016). One may adopt this strategy when detailed information about species is unavailable, making it challenging to meet all IUCN criteria for assessing their conservation status. Thus, we calculated the area occupied by the species given current and future scenarios to determine the distribution dynamics of adequate areas for the studied species and assess their extinction risk. We assigned to each climate scenario a threat category from the IUCN and Natural Resources (IUCN, 2019; Thuiller et al., 2005; Akçakaya et al., 2006; Rodríguez et al., 2015). Following the IUCN Red List criterion A3(c), we use the following thresholds for each threat category: Extinct (EX): 100% projected area loss within a maximum of 100 years; Critically Endangered (CR): ≥ 80% area loss; Endangered (EN): area loss <80% and ≥ 50%; Vulnerable (VU): area loss <50% and ≥ 30%; Near Threatened (NT): loss <30%. Despite being a simplistic approach and taking into account only the effects of climate change, it can provide us with an overview of the threats for each species individually, thus being vital for decision-makers in conserving these species. We performed all analyses in R software v4.0.3 (R Core Team, 2020) using the raster package (Hijmans, 2015).

## Results

### Species distribution models

We built SDMs for 19 bird species from the Caatinga domain. The values for model evaluation (Jaccard) varied between 0.63 (*Herpsilochmus sellowi*) and 0.96 (*Anodorhynchus leari,* see Table S1). Sixteen out of the 19 studied species will lose suitable occurrence areas under RCP4.5, and 17 will lose areas under RCP 8.5. The number of highly vulnerable species, which lost more than 40% of their original ranges, was six and 11 in RCP 4.5 and RCP 8.5 scenarios, respectively (Table S2). In RCP 4.5, *Anodorhynchus leari* (62%) had the most significant area loss. *Pyrrhura griseipectus* faced a reduction of 91% of its original distribution in RCP8.5 (see Fig. S1 for maps of each species). On the other hand, our results showed that suitable areas would expand for three species in RCP4.5: *Nyctidromus hirundinaceus, Penelope jacucaca,* and *Xiphocolaptes falcirostris*, with gains of 0.2%, 3%, and 15% in their geographical ranges, respectively. *Penelope jacucaca,* and *Xiphocolaptes falcirostris*, in turn, expanded their ranges by 2% and 14%, respectively, in RCP8.5 (see Fig. S1).

### Species richness and stable areas

**Figure 2:**
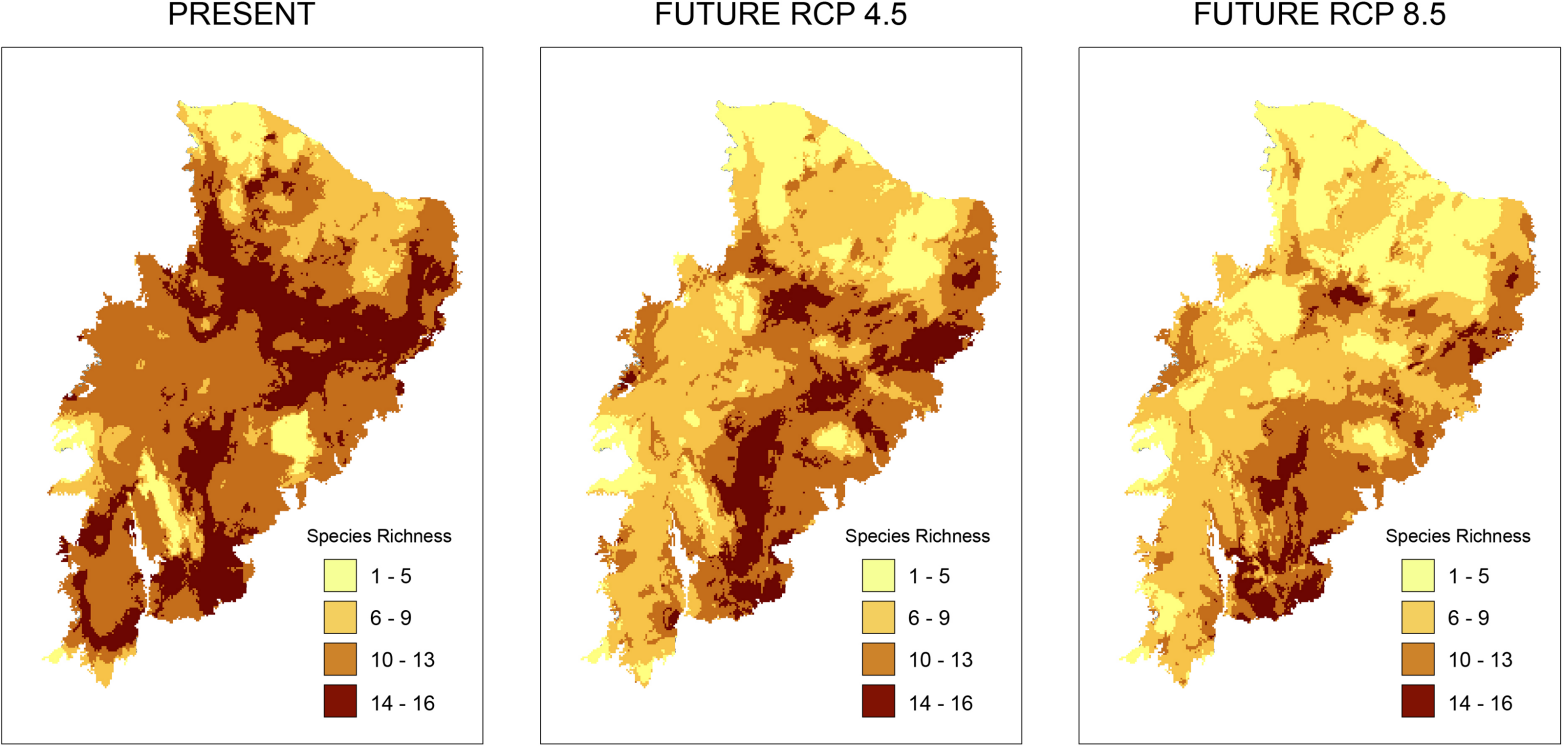
Richness maps of endemic and/or endangered bird taxa in the Caatinga domain. Richness maps of endemic and/or endangered bird taxa in the Caatinga domain for both present and future (RCP 4.5 and RCP 8.5) generated from stacking distribution models of all species. Darker areas correspond to areas where there is higher taxa richness. Shapefile of the domain provided by MMA (Ministério do Meio Ambiente do Brasil-Ministry of the Environment).

According to the models for the current scenario, the regions with the highest taxonomic richness are located in a small part of the Caatinga where few PAs exist. Analyzing taxonomic representativeness within the PAs in future scenarios, we observed a loss of areas with high species richness (Fig. 2). Areas of high richness harbor between 13 and 16 species in both present and future predictions. However, we notice a significant decrease in species numbers in the richest areas. This was especially true in the RCP 8.5 projection, where areas became smaller and more sparsely distributed (Fig. 2). A decrease in richness from the present to the two future scenarios is also observed for the other species richness ranges (Fig. 2).

**Figure 3:**
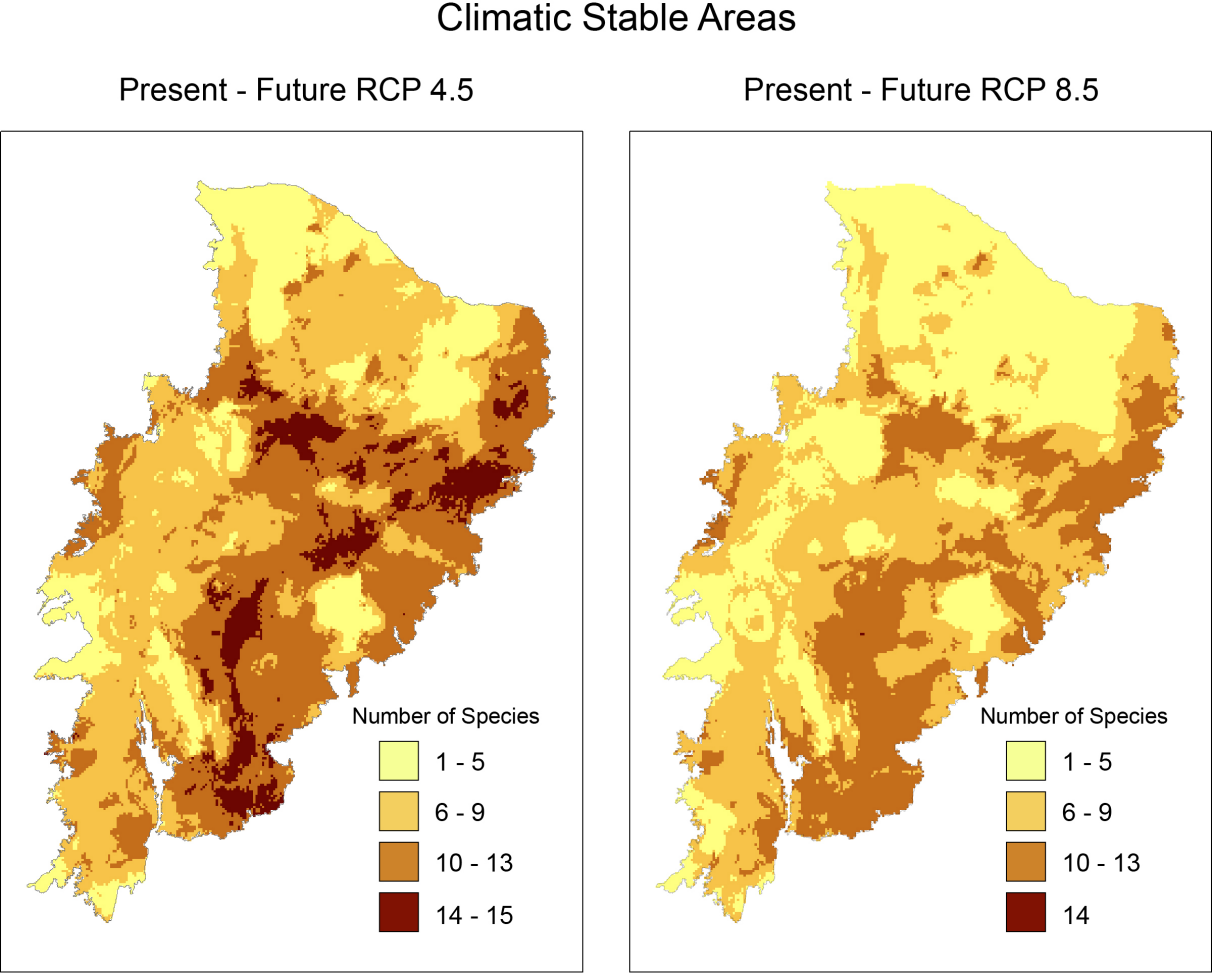
Richness map of stable areas with endemic and/or endangered bird taxa in the Caatinga domain by stacking stable areas of all species. Darker areas correspond to areas with higher taxa richness. Shapefile of the domain provided by MMA (Ministério do Meio Ambiente do Brasil-Ministry of the Environment.

In climatically stable areas, in the overlapping ranges of the species, we observed that there are minimal ranges, for the maximum range of overlap (13–16 species), in the two future scenarios, especially in RCP 8.5, where we observed only 42 km^2^ of range (two cells) (Fig. 3). In the stable area in the RCP4.5 projection, we detected the presence of few PAs: Chapada do Araripe Environmental Protection Area (APA), APA Serras e Brejos do Capibaribe, APA Ambiente das Onças, APA, Boqueirão da Onça, APA Marimbus-Iraquara, Parque Nacional do Catimbau, Estação Ecológica Raso da Catarina, Parque Estadual do Morro do Chapéu, Floresta Nacional de Contendas do Sincorá, Terra Indígena Xukuru (TI), TI Pipipã, TI Kambiwá, TI Brejo do Burgo, TI Pankararé. There was no PA in the stable area with a high overlapping range of species for RCP8.5. We also verified that the range size of overlapping species considerably decreased from RCP4.5 to RCP8.5. In the following range of overlap (9–13 species) are all PAs encompassed in the maximum range of RCP4.5 with the addition of the Serra das Confusões National Park.

### PA effectiveness and conservation status of species

In the current scenario, the gap analysis including only SPAs showed that no species was considered protected and one specie is as unprotected (Table 1). Therefore, SPA areas alone cannot effectively protect the bird species analyzed here. Considering the SPA+SUA, no species could be regarded as protected. Seventy-eight percent of species were partially protected, 15% corresponded to gaps, and 5% were not protected. Finally, in the broader protection scenario (SPA+SUA+IL), only one species was considered protected; 73% were partially protected, 15% remained within the gap category, and 5% were not protected.

**Table 1:** Effectiveness of protected areas and conservation status of species. Summary with the number of Caatinga bird species classified in each protection category and level of Protected Areas in the gap analysis for present (current) and futures scenarios (RCP 4.5 and RCP 8.5). Strictly Protection Areas (SPA), Sustainable Use Areas (SUA), and Indigenous lands (IL).

	Present			Future 4.5			Future 8.5		
	SPA	SPA +SUA	SPA +SUA+IL	SPA	SPA +SUA	SPA +SUA+IL	SPA	SPA +SUA	SPA +SUA+IL
Protected (P)	0	0	1	0	0	1	0	0	0
Partially Protected (PP)	2	15	14	6	14	13	5	14	14
Gaps (G)	16	3	3	11	4	4	12	4	4
Not protected (NP)	1	1	1	2	1	1	2	1	1
Total	19	19	19	19	19	19	19	19	19

In future scenario RCP4.5, where only SPA areas were considered, no species met the criteria of the protected status (Table 1). Likewise, the gap analysis, including SPAs, shows that no species met the requirements to be protected (Table 1), while 5% of species were not protected. Considering the SPA+SUA scenario, no species is considered protected, 73% are partially protected, 21% are gap, and 5% fall into the non-protected status. In the broader protection scenario (SPA+SUA+IL), only one species was protected; 68% were partially protected, 21% remained as gaps, and 10% were not protected. In future scenario RCP8.5, where only SPA areas were considered, no species reached the protected status (Table 1), and 10% of species were not protected. Considering the SPA+SUA scenario, none of the species was protected; 73% were partially protected, 21% were gaps, and 5% were not protected. Lastly, in the broader protection scenario (SPA+SUA+IL), we did not find any protected species; 73% are partially protected, 21% fell into the gap category, and 5% were not protected.

The species *A. leari*, *Lepidocolaptes wagler,* and *P. griseipectus* in all scenarios and all categories of PAs were considered as gap species. In contrast, *Sclerurus cearensis* is considered a gap only in future scenarios regardless of the categories of PAs. *Rhopornis ardesiacus* in current and future, and all PAs categories met the criteria of a not protected species. Interestingly, these four species are currently classified as Endangered (EN) by the IUCN.

Interestingly, ineffective PAs in the current period remained ineffective in 2070. Under future climatic conditions, for RCP 4.5, we remain with <9% of PAs considered ineffective, while in RCP 8.5, this value decreased to 6% (Table S3).

The MPO between the distribution of bird species and the network of protected areas (PAs) in the Caatinga revealed inadequate protection. In the current scenario, the average percentage of overlap was 0.11% (ranging from 0.02 to 0.15%) (Table S3). In the future, the average percentage of overlap was 0.11% (ranging from 0.04 to 0.13%) for RCP 4.5 (Table S3) and 0.09% (ranging from 0.01 to 0.14%) for RCP8.5 (Table S3). The MPO relationship evidenced a more precarious conservation scenario for species within and below the random range. The protection of existing PAs was respectively equal to or worse than expected by a random distribution of the existing PAs (Table S3). Within the scenario of poor protection of Caatinga bird species, none had an MPO value above the expected by chance in the present and the future, representing 52.63%, 21.05%, and 21.05% of the species presented MPO not significantly different from the expected by chance in the current and future scenarios, respectively (Table S3). In even less favorable conditions, 47.36%, 78.94%, and 78.94% of bird species in the Caatinga were less representative than expected in current and future scenarios (RCP 4.5 and RCP 8.5), respectively (Table S3).

According to IUCN criterion A3(c), for the RCP 4.5 scenario, 42% of Caatinga bird species and 68% of species are projected to be threatened in RCP 8.5 by 2080 (Table S2). Our results also indicated that for RCP 4.5, four species were classified as EN and four as VU. For RCP 8.5, there will be three CR, four EN, and six VU species. *A. leari, P. griseipectus,* and *S. cearenses* had the most significant impact on their threat status, especially the RCP 8.5 scenario, classified as CR species. A summary of the impacts of future climate change on the conservation status of each species is shown in Table S2.

## Discussion

We observed that 84% and 87% of the bird species of Caatinga analyzed in this study will lose significant portions of their predicted range distributions in the RCP4.5 and RCP8.5 future scenarios, respectively. Still, in RCP8.5, the most pessimistic scenario, species will lose more areas than in RCP 4.5. Additionally, we observed that the current PAs network design in the Caatinga was ineffective in protecting endemic and endangered bird species in current or future climatic scenarios, even when all PAs were considered.

Our results show an apparent reduction in the number of suitable areas for most species in response to climate changes, which corroborates a pattern already observed for other taxonomic groups (Caten et al., 2017; Collevatti et al., 2012; Loyola et al., 2012; Terribile et al., 2012). This pattern probably reflects the effect of shifts in rainfall regimes, which might be more drastic in tropical regions (Moura & Hastenrath, 2004; Nobre et al., 2007). The loss of suitable habitat due to projected future climate change for species of STDFs is already well known. Silva et al. (2019) showed that, throughout the Caatinga biome, areas of suitable habitat for endemic plant species would be reduced under both optimistic and pessimistic future climate change scenarios. Simoes et al. (2019) found that endemic cacti species are threatened with extinction due to the retraction of environmentally suitable areas. This area reduction for plants in STDFs was also reported in other works (Oyama & Nobre, 2003; De Andrade et al., 2017, Marengo, Torres & Alves, 2017). Garcia, Ortega-Huerta & Martinez-Meyer (2013) suggested an average reduction of about 64% in the geographic range of all endemic amphibians in Mexican STDFs by the year 2080 as a consequence of climate change. Peterson et al. (2002) revealed a reduction of up to 50% in the current distribution of 20% of 1870 Mexican mammals, birds, and butterflies until the year 2050. Prieto-Torres et al (2020) investigated birds from the dry forests of Central and South America and found that 75% of the bird species in these forests will face a reduction of climatically suitable areas.

The birds experiencing the highest reduction in suitable areas (over 60%), *A. leari, P. griseipectus, Rhopornis ardesiacus,* and *Sclerurus cearensis*, were species having a restricted distribution and highly associated with specific microhabitats. Despite the great effort toward protecting *Rhopornis ardesiacus*, *e.g.*, by creating a National Park and a Wildlife Refuge in Boa Nova (Bahia, Brazil), this species is still losing its natural habitat due to deforestation in other areas where it occurs. Aside from the strong anthropic pressure and area loss due to climate changes, *R. ardesiacus* is suffering from increased luminosity inside forests, which decreases food availability and reproductive resources for this species. This higher luminosity causes a decrease in the vegetative growth of bromeliads of the genus *Aechmea*, with which *R. ardesiacus* closely interacts (Luiz, 2010; Villegas, 2006; Luiz et al., 2015). Moreover, besides facing significant area loss (40% and 60%, RCP 4.5 and RCP 8.5, respectively), this species was found under the not protected category in all situations assessed herein.

The *Pyrrhura griseipectus* is also predicted to undergo a high loss of suitable areas (60% and 91%, RCP 4.5 and RCP 8.5, respectively). It can be found in “brejos de altitude” and dry forests destroyed due to property speculation, with only 13% of its original area remaining (Albano & Girão, 2008; Girão, Albano & Campos, 2010). Moreover, illegal trade of individuals and deforestation of its natural habitats are the main threats to this bird. Our results reinforce the widely accepted idea that significant changes in the biota’s spatial ranges of dry forests will occur during the 21st century (Golicher, Cayuela & Newton, 2012; Rojas-Soto, Sosa & Ornelas, 2012; Collevatti et al., 2012; Prieto-Torres et al., 2016; Silva et al., 2019). Species in forested habitats in the Caatinga will suffer a more significant reduction than the species of the “Depressão sertaneja.” Thus, it seems plausible that species will track their climatic niches dispersing to higher areas, where they can find similar climatic conditions to their current distributions (Meir & Pennington, 2011; Prieto-Torres & Rojas-Soto, 2016). Potential migrations to higher altitudes could produce local extinctions or contractions in the spatial distribution of habitat specialists and species with small ranges. Species that live in more elevated areas would face the most significant challenges because they might not be able to move to colder areas since they do not exist in the Caatinga (Pacifici et al., 2017).

The unique climatic conditions of Caatinga have caused adaptive singularities in its biota, which has led to the development of unique physiological adaptations and specific reproductive behaviors, thus showing higher plasticity in their ecological traits (Rodrigues, 1996; Rodrigues, 2003; Vieira, Santana & Arzabe, 2009). Although species are inserted and adapted to the current climatic conditions in the Caatinga and have been shaped during their evolutionary history, they might not have the intrinsic ability to adapt to climate change of the magnitude predicted for the future at a short temporal scale. In tropical regions, tropical species already experience not only climatic conditions close to their physiological tolerance limits but also faster rates of climate change (Deutsch et al., 2008; Domingos et al., 2014). Thus, exceeding these climatic limits can reduce their ability to cope with changes in climate (Huey et al., 2012; Kingsolver, Diamond & Buckley, 2013; Khaliq et al., 2014). Khaliq et al. (2014) found in their studies that tropical endotherms have narrower thermal safety margins and were already experiencing maximum temperatures close to their thermal limits. However, this tolerance varies among bird orders. For example, Caprimulgiformes appear to have higher heat tolerance limits than Passeriformes (McKechnie et al., 2016; McKechnie et al., 2017; Smit et al., 2018; Albright et al., 2017; Pollock et al. , 2021). This seems to be compatible with our result since the geographic range of *Nyctidromus hirundinaceus* (Caprimulgiformes) barely changed in our projections for the future.

### Protecting areas in the current and the future scenarios

Richness in all investigated scenarios and stable areas maps suggest essential areas for the biome’s southern, central and eastern regions. The southern portion of Caatinga is not currently protected. However, it is an area of high species richness and has been identified as a high-priority biodiversity conservation spot by the Brazilian Ministry of the Environment (MMA, 2021). Furthermore, this region is home to sites of interest for establishing new areas of integral protection due to extensive natural remnants (MMA, 2021; Antongiovanni, Venticinque & Fonseca, 2018; Antongiovanni et al., 2020). The south of the Caatinga is fundamental for the conservation of cacti species (Carvalho et al., 2022) and one of the few areas in eastern Brazil capable of safeguarding different Cactaceae species in climate change scenarios in the next 60 years (Simoes et al., 2019). The east and central parts are also crucial for conserving endemic plants from the Caatinga dry forest (Silva et al., 2019), corroborating the pattern found in the present study. In our predictions, we found that the richest site is located in the central region of the domain. However, the natural vegetation of this area is not only currently highly fragmented (Antongiovanni, Venticinque & Fonseca, 2018) but also is very susceptible to desertification (Salazar, Nobre & Oyama, 2007) due to the high temperatures, decreased rainfall, soil degradation, and their combination (Darkoh, 1998; Geist & Lambin, 2004; Sivakumar, 2007).

Although protected areas provide an essential service in protecting species, their current configuration in the Caatinga is inefficient for conserving the bird species studied herein, even considering all protection categories (PI+US+IL). Most protected areas we assessed tend to protect areas of low species richness, and just a few PAs protected high species numbers. The role of PAs in protecting species has been discussed regarding several taxa in different domains (Lemes, Melo & Loyola, 2014; Carvalho et al., 2017; Oliveira et al., 2017). For instance, PAs do not preserve restricted/rare Odonata species in the Cerrado (Nóbrega & De Marco, 2011). PAs in the Amazon do not fully protect fish biodiversity in watercourses. Prieto-Torres et al (2018), when evaluating the PAs of several neotropical dry forests, found that the current network covers less than 15% of the distribution ranges of 80% of bird species. The authors also detected that it would be necessary to double the area enclosed within the PAs so that their biodiversity could be more efficiently sheltered. The ineffectiveness of PAs in Caatinga might be directly related to their small number and size.

Another issue that has been discussed is which types of PAs are more effective for biodiversity conservation: more restricted use sites (*e.g.*, National Parks, Biological Reserves, Ecological Stations) or sustainable use units (*e.g.*, National Forests, Extractive Reserves; Locke & Dearden, 2005; Sims, 2010; Ferraro et al., 2013; Carranza et al., 2014). Teixeira et al. (2021) found that fully protected PAs reduce deforestation within their limits. This author also emphasized the ineffectiveness of sustainable use of PAs, especially the ones classified as “Áreas de Proteção Ambiental” (APAs). The same issue has already been reported in other domains such as the Cerrado (Carranza et al., 2014; Françoso et al., 2015; Paiva, Brites & Machado, 2015) and the Amazon (Nepstad et al., 2006; Adeney, Christensen Jr & Pimm, 2009; Soares-Filho et al., 2010; Nolte et al., 2013).

PAs cover 9% of the Caatinga region (Brasil, 2020); only 1.8% are fully protected areas, while 7.2% consist of sustainable use areas, where the direct use of natural resources is permitted by law (Brasil, 2020). According to our gap analysis, it is worth noting that Sustainable Use PAs are of great importance since they partially protect our target species. Thus, it becomes essential to reassess the protection category of these PAs, given that sustainable use areas are not quite efficient for containing human disturbances, as we previously mentioned (Antongiovanni, Venticinque & Fonseca, 2018; Antongiovanni et al., 2020). Most of the Caatinga PAs face problems related to administrative management, such as the absence of land tenure regularization and the lack of a management plan. Around 92.5% of the Caatinga protected areas do not only have a management plan (Brasil, 2020) but suffer from both the lack of human resources and financial support (Drummond, Franco & Ninis, 2009). Along with deforestation, PAs also regularly face synergetic, agricultural, cattle grazing, and burning activities (Feliciano et al., 2003; CSR/IBAMA, 2014).

Therefore, it is crucial to evaluate the role of protected areas in adequately protecting Caatinga’s biodiversity. It is equally important to note that, in addition to climatic conditions, the long-term viability of the endemic bird species of Caatinga will also depend on the persistence of vegetation remnants (which still exist). That is, preserving those remnants still within PAs and creating ecological corridors between the external fragments to make it possible for the species to move across the landscape. In this regard, conservation efforts should be directed towards expanding current PAs, possibly changing the protection category of PAs, and implementing priority areas for conservation proposed by the MMA. In addition, the creation of ecological dispersion corridors within stable areas to avoid population isolation and increase their viability under climate change should be considered (Beier & Noss, 1998).

It is also worth highlighting that all these measures would help us achieve the biodiversity conservation goals proposed in 2013 by the National Biodiversity Commission (Conabio, 2013). One of these proposals was to improve the Brazilian biodiversity conservation status by creating new PAs and other protected areas to protect at least 30% of the Amazon and 17% of the other terrestrial biomes, including the Caatinga (Brasil, Ministério do Meio Ambiente, 2013). However, only 9% of the Caatinga territory is covered by protected areas, and none of our studied species are under the protection of the current PAs. Lastly, we also emphasize the need for environmental education initiatives to increase awareness of the locals about the importance of both biodiversity and PAs.

## Conclusions

In this study, we verified the inefficiency of the PAs in protecting the endemic bird species of Caatinga. Still, despite showing the significant vulnerability of the species to climate change, our results also show us possibilities to increase the viability of these species in the future. Here, we offer possibilities to direct efforts toward maintaining and implementing ecological corridors and expanding PAs.

##  Supplemental Information

10.7717/peerj.14882/supp-1 Supplemental Information 1 Raw data Geographic coordinates

10.7717/peerj.14882/supp-2 Supplemental Information 2 Supplemental Tables and Figure

## Additional Information and Declarations

### Data Availability

The following information was supplied regarding data availability: The raw data are available in the Supplemental Files.
