# Supplementary material for: Multi-temporal ecological niche modeling for bird conservation in the face of climate change scenarios in Caatinga, Brazil
Source: PeerJ. 2023 Feb 27;11:e14882. doi: 10.7717/peerj.14882 (PMC9979838; doi:10.7717/peerj.14882)

SUPPORTING INFORMATION

**Multi-temporal ecological niche modelling for bird conservation in the face of climate change scenarios in Caatinga, Brazil**

Gabriela Silva Ribeiro Gonçalves*, Pablo Vieira Cerqueira, Daniel Paiva Silva, Letícia Braga Gomes, Camila Ferreira Leão, André Felipe Alves de Andrade, Marcos Pérsio Dantas Santos

***corresponding author: gabrielasrgoncalves@gmail.com**

**Table S1.** List of 19 bird taxa selected to be used in the Species Distribution Models, List of 19 birds endemic of Caatinga, used to build species distribution models, with final number of occurrences, Receiver Operationg Characteistic threshold (TSH) and values Jaccard. Asterik (*) indicate endemic and near-endemic taxa.

| Species | Unique occurrences | TSH | Jaccard |
| --- | --- | --- | --- |
| *Anodorhynchus leari** | 17 | 0.76 | 0.96 |
| *Anopetia gounellei** | 68 | 0.62 | 0.65 |
| *Crypturellus zabele** | 42 | 0.57 | 0.86 |
| *Herpsilochmus sellowi** | 145 | 0.63 | 0.63 |
| *Hylopezus ochroleucus** | 62 | 0.87 | 0.83 |
| *Lepidocolaptes wagleri* | 24 | 0.43 | 0.92 |
| *Megaxenops parnaguae** | 93 | 0.66 | 0.73 |
| *Myrmorchilus strigilatus* strigilatus* | 204 | 0.51 | 0.70 |
| *Nyctidromus hirundinaceus** | 137 | 0.68 | 0.78 |
| *Penelope jacucaca** | 62 | 0.56 | 0.76 |
| *Pyrrhura griseipectus* | 8 | 0.68 | 0.81 |
| *Rhopornis ardesiacus** | 6 | 0.63 | 0.76 |
| *Sakesphorus cristatus** | 184 | 0.71 | 0.73 |
| *Sclerurus cearensis* | 31 | 0.82 | 0.74 |
| *Spinus yarrellii* | 42 | 0.58 | 0.73 |
| *Stigmatura budytoides gracilis** | 99 | 0.67 | 0.81 |
| *Stigmatura napensis bahiae** | 136 | 0.84 | 0.85 |
| *Synallaxis hellmayri** | 113 | 0.77 | 0.71 |
| *Xiphocolaptes falcirostris* | 50 | 0.54 | 0.88 |

**Table S2.** List of birds species of Caatinga used to build species distribution models with the summarization of the climate change effects on species distribution. Estimated area size for each scenario (Present and Future) and the area variation between present and future scenarios represent reduction of estimated areas when percentage values were positive and expansion when negatives, and classification of criterian A3(c) of IUCN.

| Species | Estimated area Present (km²) | Estimated area RCP4.5 (km²) | Estimated area RCP8.5 (km²) | | % of lost (-) and gained (+) of cells (Present-RCP 4.5) | % of lost (-) and gained (+) of cells  (Present-RCP 8.5) | | IUCN  (Present) | IUCN Criterian  A3(c) (RCP4.5) | IUCN Criterian A3(c) (RCP8.5) |
| --- | --- | --- | --- | --- | --- | --- | --- | --- | --- | --- |
| *Anodorhynchus leari* | 33350.94 | 12530.7 | | 6297.48 | -62.43 | | -81.11 | EN | EN | CR |
| *Anopetia gounellei* | 812075.0 | 430991.8 | | 309369.1 | -46.93 | | -61.90 | LC | VU | EN |
| *Crypturellus zabele* | 511809.5 | 492060.2 | | 446285.7 | -3.86 | | -12.80 | Not Evaluated | NT | NT |
| *Herpsilochmus sellowi* | 796588.4 | 460937.0 | | 352830.2 | -42.14 | | -55.71 | LC | VU | EN |
| *Hylopezus ochroleucus* | 625421.2 | 496065.8 | | 366710.4 | -20.68 | | -41.37 | NT | NT | VU |
| *Lepidocolaptes wagleri* | 133532.3 | 105986.2 | | 75869.6 | -20.63 | | -43.18 | Not Evaluated | NT | VU |
| *Megaxenops parnaguae* | 619230.8 | 254726.6 | | 154716.7 | -58.86 | | -75.01 | LC | EN | EN |
| *Myrmorchilus strigilatus strigilatus* | 810104.4 | 806355.9 | | 792947.0 | -0.46 | | -2.12 | LC | NT | NT |
| *Nyctidromus hirundinaceus* | 771762.6 | 773454.8 | | 767542.9 | 0.22 | | -0.55 | LC | NT | NT |
| *Penelope jacucaca* | 785835.5 | 811646.6 | | 804363.8 | 3.28 | | 2.36 | VU | NT | NT |
| *Pyrrhura griseipectus* | 90885.06 | 36028.44 | | 7411.32 | -60.35 | | -91.84 | EN | EN | CR |
| *Rhopornis ardesiacus* | 11266.92 | 6790.14 | | 4455.36 | -39.73 | | -60.45 | EN | VU | EN |
| *Sakesphorus cristatus* | 831160.3 | 705382.0 | | 557734.0 | -15.13 | | -32.90 | LC | NT | VU |
| *Sclerurus cearensis* | 186761.0 | 82595.5 | | 31208.9 | -55.77 | | -83.29 | VU | EN | CR |
| *Spinus yarrellii* | 374443.0 | 336872.3 | | 240460.9 | -10.03 | | -35.78 | VU | NT | VU |
| *Stigmatura budytoides gracilis* | 695893.0 | 602137.6 | | 543725.3 | -13.47 | | -21.87 | LC | NT | NT |
| *Stigmatura napensis bahiae* | 609291.9 | 431484.5 | | 341670.4 | -29.18 | | -43.92 | LC | NT | VU |
| *Synallaxis hellmayri* | 674365.9 | 451105.2 | | 341970.3 | -33.11 | | -49.29 | LC | VU | VU |
| *Xiphocolaptes falcirostris* | 672073.9 | 774054.5 | | 771591.2 | 15.17 | | 14.81 | VU | NT | NT |

**Table S3.** Mean percentage of spatial overlap (MPO) between the distribution areas of the birds species and protected areas of the Caatinga at present and future (2070) times and results of null models describing the representativeness of species in protected areas: (+) significantly higher than expected by chance, (-) significantly lower than expected by chance and (*) non-significant (*p*<0.05).

|  | Present | | | Future RCP4.5 | | | Future RCP8.5 | | |
| --- | --- | --- | --- | --- | --- | --- | --- | --- | --- |
| Species | MPO | MPO randomizad | Representativeness | MPO | MPO randomizad | Representativeness | MPO | MPO randomizad | Representativeness |
| Sp1 | 0.103404 | 0.124912 | - | 0.071795 | 0.1258188 | - | 0.027211 | 0.12550907 | - |
| Sp2 | 0.124341 | 0.125217 | * | 0.100343 | 0.1252461 | - | 0.096933 | 0.12519634 | - |
| Sp3 | 0.138236 | 0.125137 | * | 0.120277 | 0.1251946 | * | 0.128198 | 0.12528764 | * |
| Sp4 | 0.118315 | 0.125225 | - | 0.098889 | 0.1251924 | - | 0.093006 | 0.12534964 | - |
| Sp5 | 0.117791 | 0.125263 | - | 0.118835 | 0.1251796 | - | 0.115006 | 0.12522882 | - |
| Sp6 | 0.131071 | 0.125095 | * | 0.107345 | 0.1252071 | - | 0.083119 | 0.12507306 | - |
| Sp7 | 0.155117 | 0.125367 | * | 0.081043 | 0.125174 | - | 0.068888 | 0.12534347 | - |
| Sp8 | 0.125117 | 0.125275 | * | 0.083754 | 0.1252494 | - | 0.047903 | 0.12514603 | - |
| Sp9 | 0.120624 | 0.12524 | - | 0.120707 | 0.1252449 | - | 0.117183 | 0.12532749 | - |
| Sp10 | 0.126012 | 0.125288 | * | 0.123456 | 0.1252481 | - | 0.119967 | 0.12526817 | - |
| Sp11 | 0.154843 | 0.12541 | * | 0.092747 | 0.1245981 | - | 0.098266 | 0.1240358 | * |
| Sp12 | 0.028517 | 0.124598 | - | 0.047319 | 0.1268392 | - | 0.019231 | 0.12580396 | - |
| Sp13 | 0.125738 | 0.125244 | * | 0.103398 | 0.1252634 | - | 0.092864 | 0.12509977 | - |
| Sp14 | 0.068242 | 0.125452 | - | 0.087915 | 0.1253932 | - | 0.100206 | 0.12558448 | - |
| Sp15 | 0.091013 | 0.125231 | - | 0.121447 | 0.1251901 | * | 0.146802 | 0.12539899 | * |
| Sp16 | 0.124938 | 0.125285 | * | 0.131301 | 0.1252144 | * | 0.134691 | 0.12520725 | * |
| Sp17 | 0.085639 | 0.125293 | - | 0.10837 | 0.1252775 | - | 0.095417 | 0.12503803 | - |
| Sp18 | 0.11835 | 0.125277 | - | 0.118898 | 0.1251505 | - | 0.090636 | 0.12534191 | - |
| Sp19 | 0.124554 | 0.125279 | * | 0.125854 | 0.1252373 | * | 0.124646 | 0.12521287 | - |

**Figure S4.** Map of occurrence records used for species modeling, present and future distribution models, and their respective areas of climate stability for all 19 studied taxa.


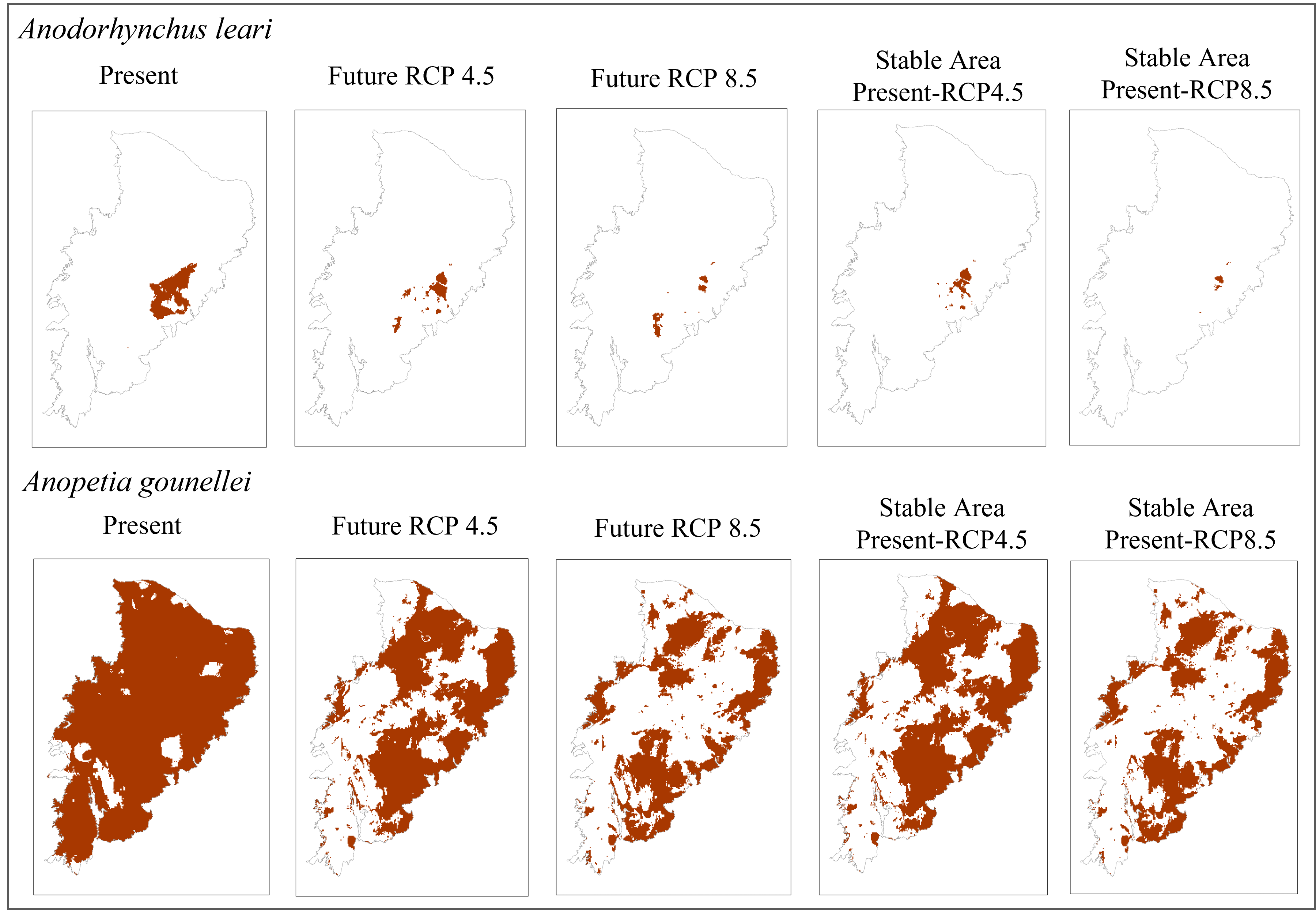


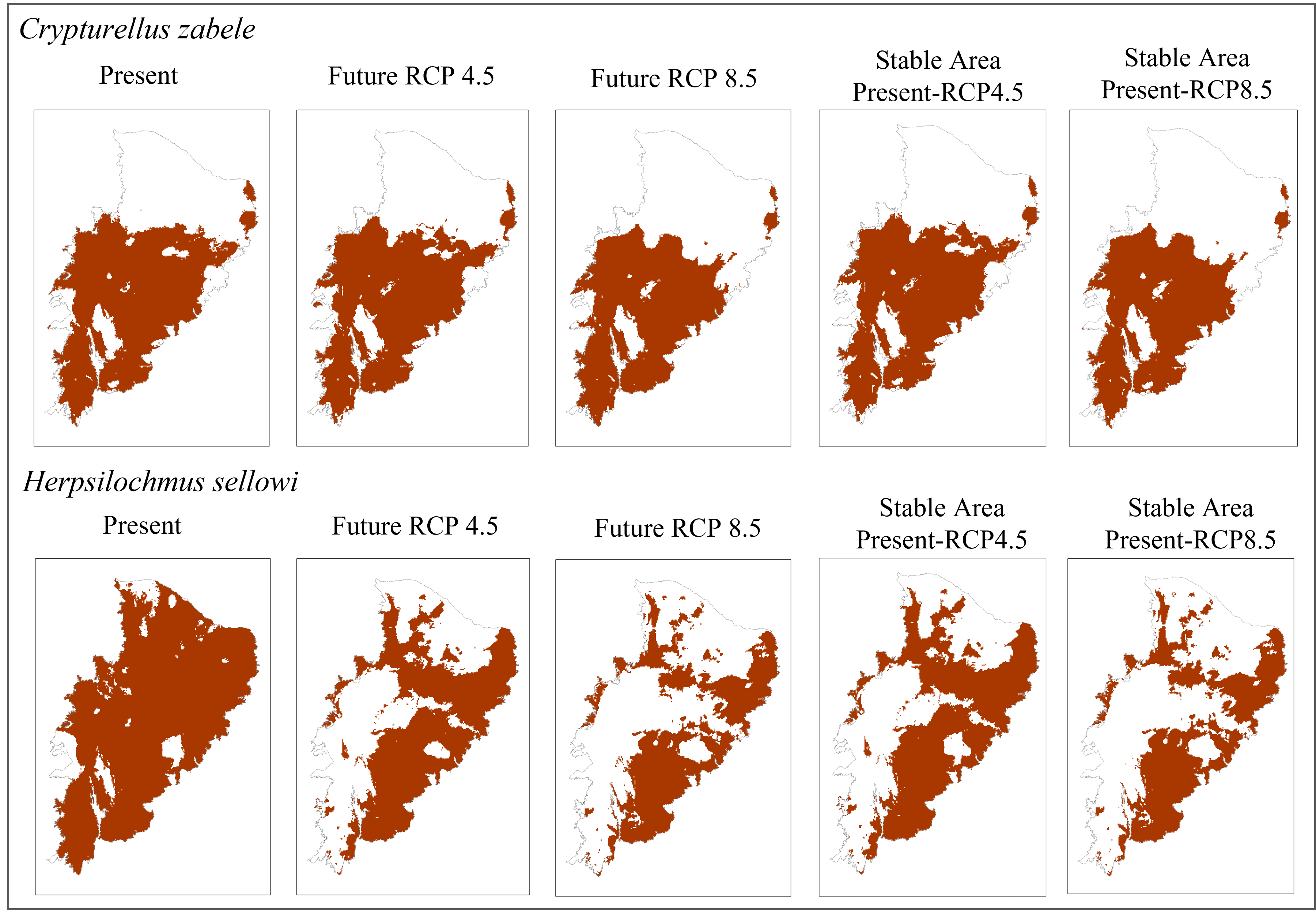


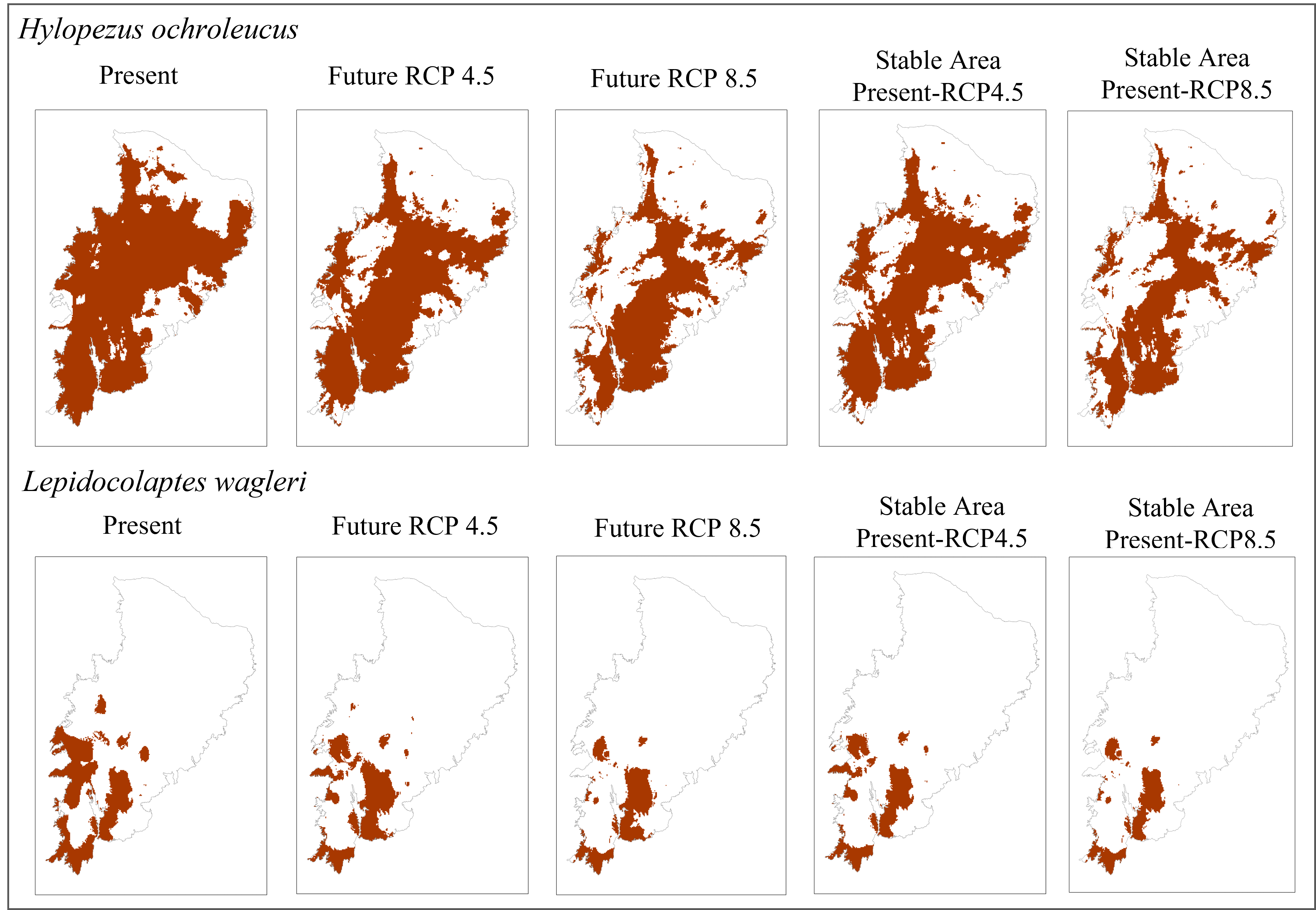


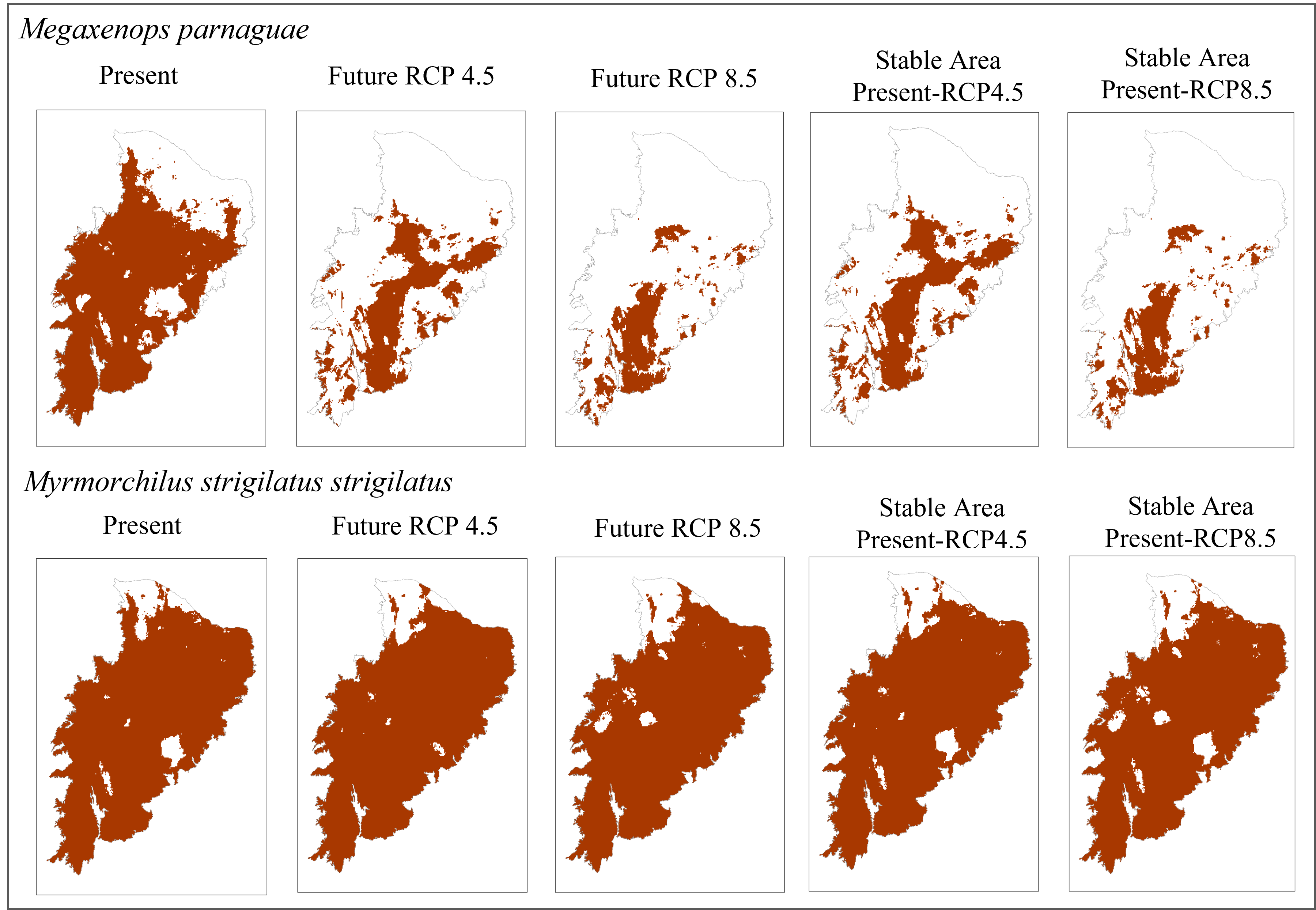


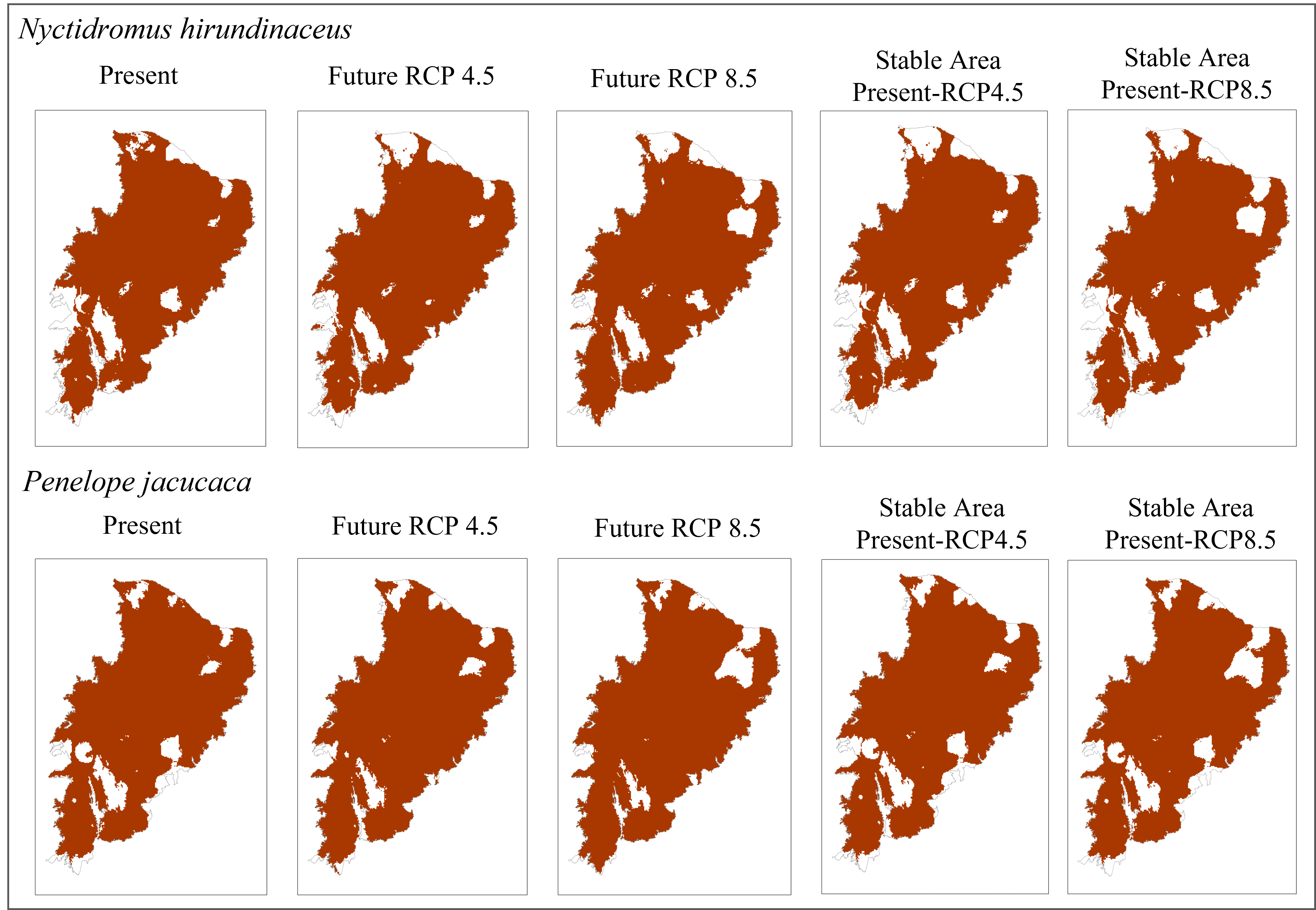


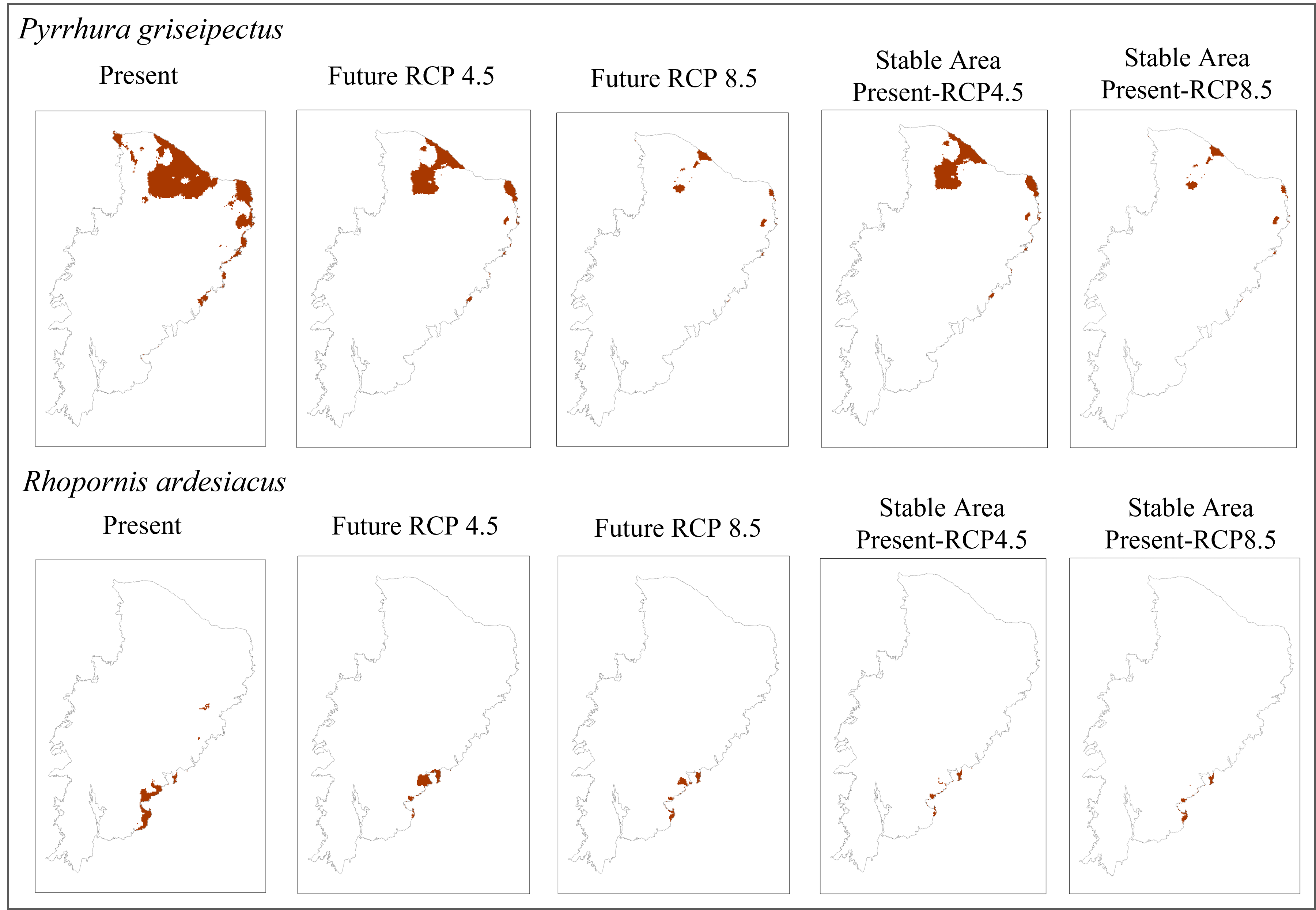


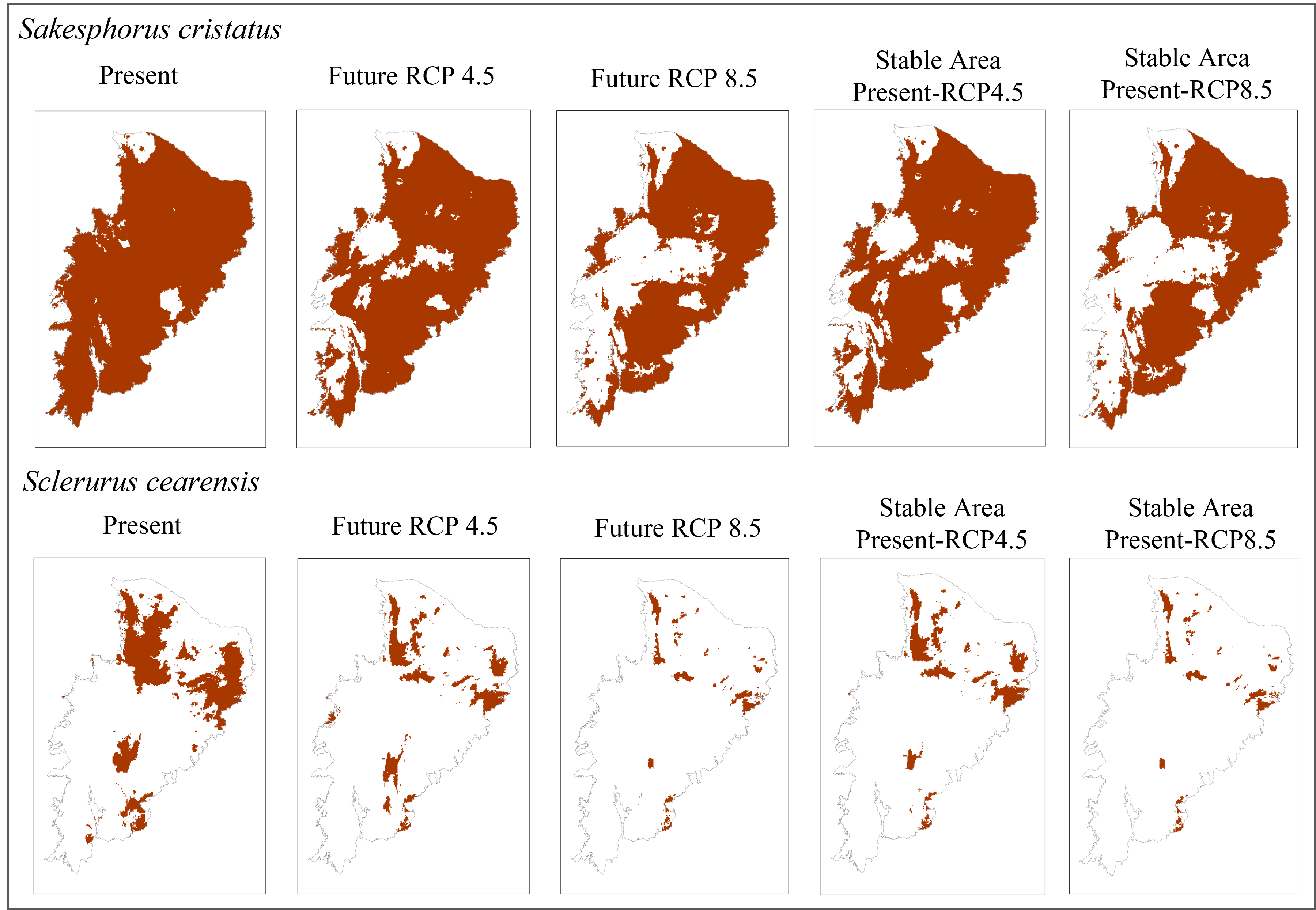


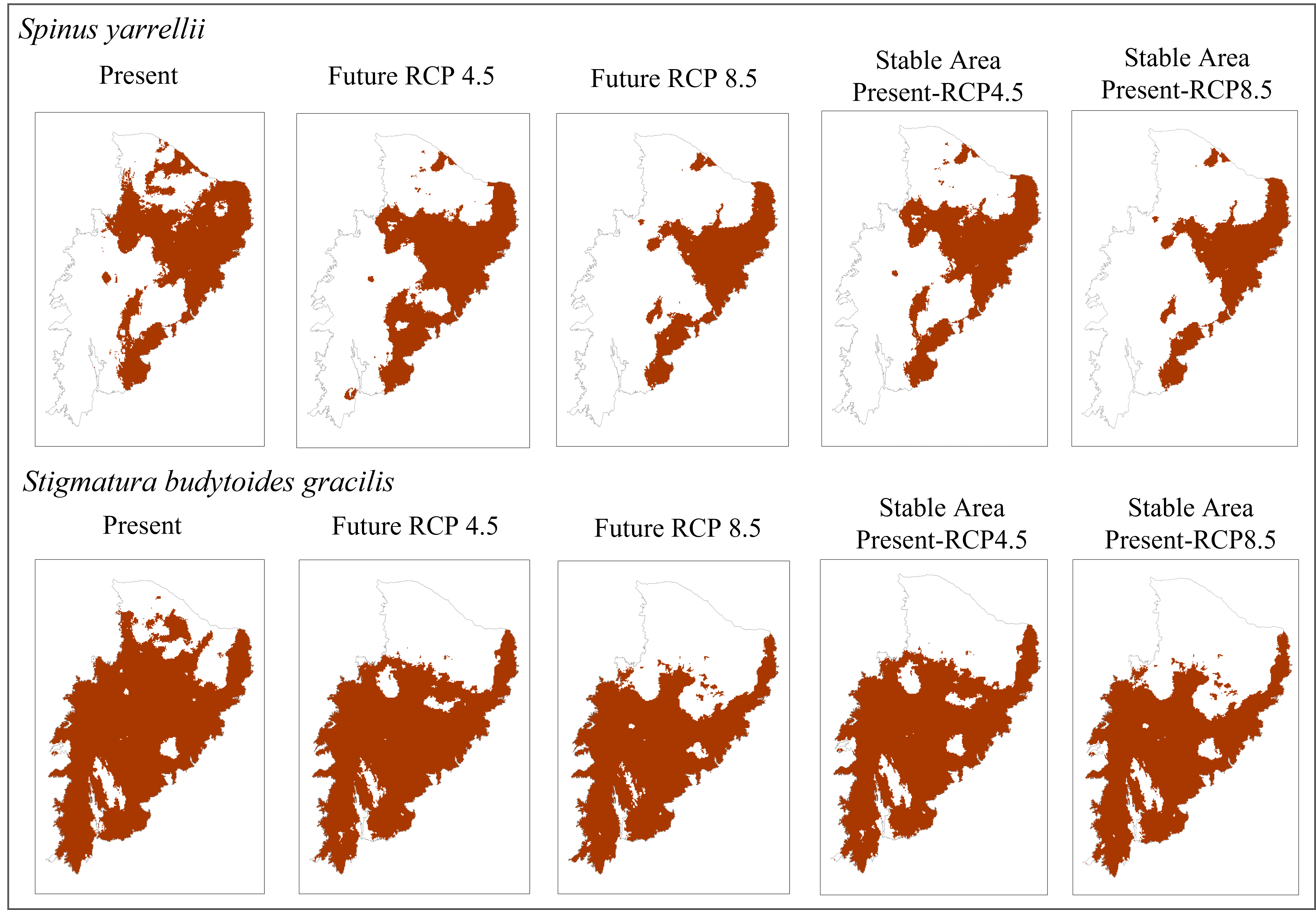


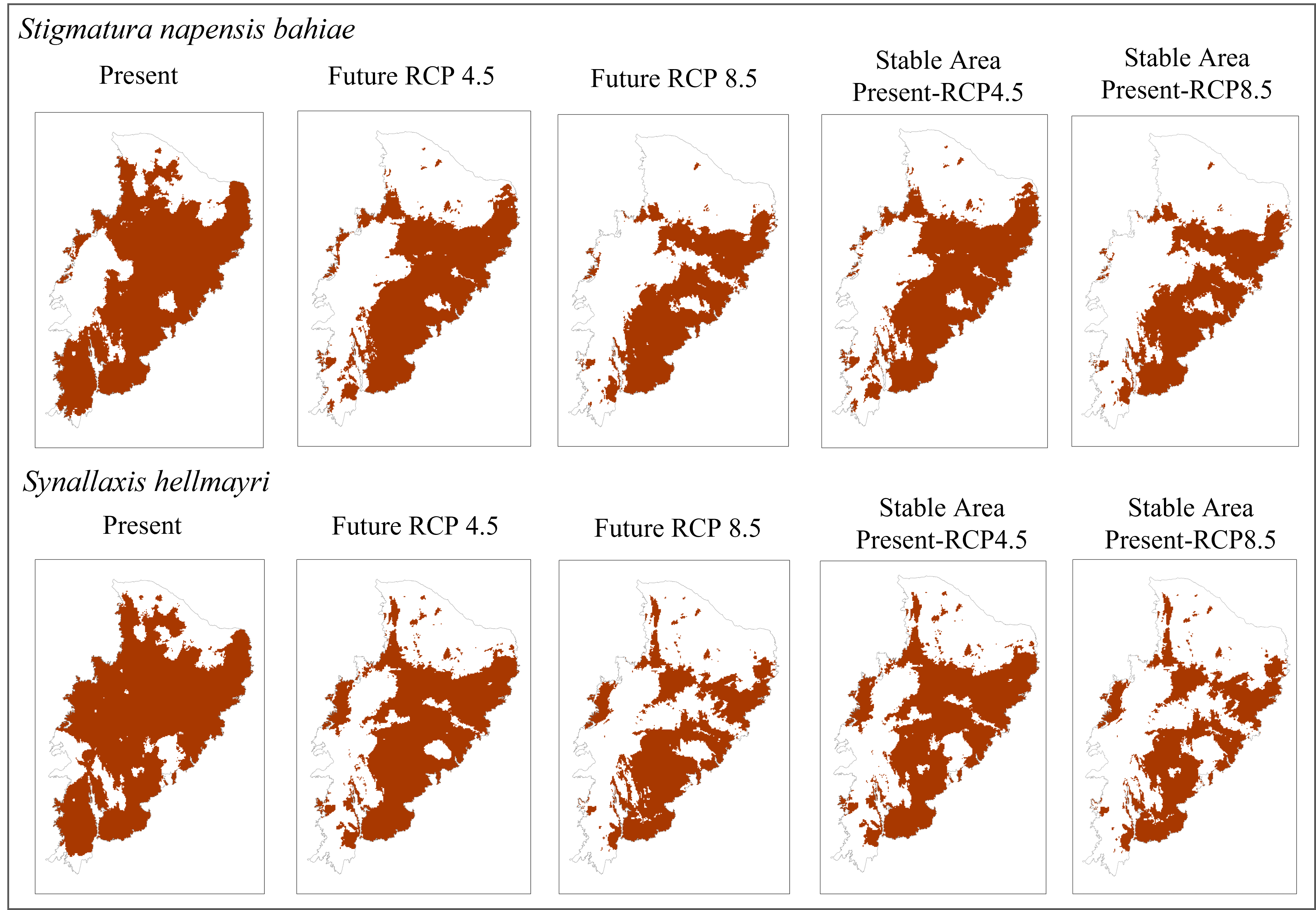


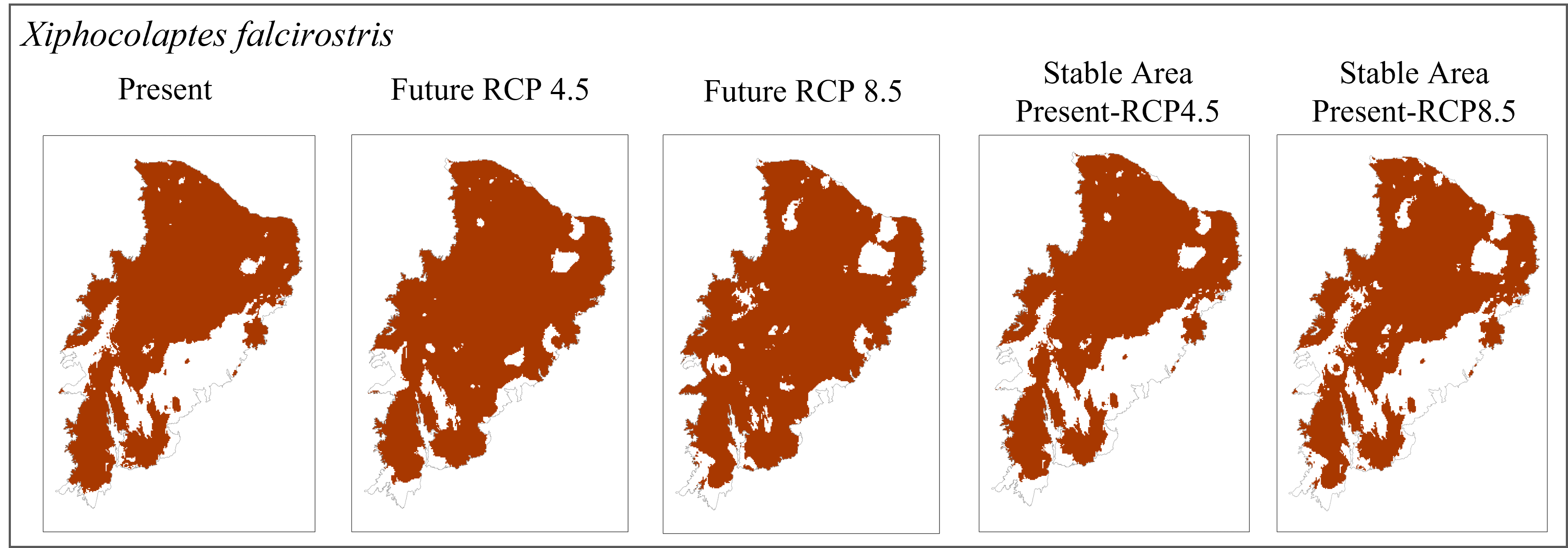

Supplement: Supplemental Information 2 [file peerj-11-14882-s002.doc]
